# The impact of ascending aorta dilatation on transcatheter aortic valve implantation outcomes

**DOI:** 10.1016/j.ijcha.2025.101680

**Published:** 2025-04-19

**Authors:** Pandit Bagus Tri Saputra, Wynne Widiarti, Ali Mustofa, Cornelia Ghea Savitri, Johanes Nugroho Eko Putranto, Faisal Yusuf Ashari, Chaq El Chaq Zamzam Multazam, Firas Farisi Alkaff, Mario D’Oria

**Affiliations:** a Department of Cardiology and Vascular Medicine, Faculty of Medicine, Universitas Airlangga, Jl. Prof. DR. Moestopo No.47, Pacar Kembang, Kec. Tambaksari, Surabaya, East Java 60132, Indonesia; b Department of Cardiology and Vascular Medicine, Dr. Soetomo General Academic Hospital, Jl. Prof. DR. Moestopo No.6-8, Airlangga, Kec. Gubeng, Surabaya, East Java 60286, Indonesia; c Faculty of Medicine, Universitas Airlangga, Jl. Prof. DR. Moestopo No.47, Pacar Kembang, Kec. Tambaksari, Surabaya, East Java 60132, Indonesia; d Faculty of Biology Medicine and Health Sciences, University of Manchester, Oxford Rd, Manchester M13 9PL, UK; e National Heart and Lung Institute, Imperial College London, Guy Scadding Building, Dovehouse St, London SW3 6LY, UK; f Department of Internal Medicine, University Medical Center Groningen, Hanzeplein 1, 9713GZ Groningen, The Netherlands; g Department of Anatomy, Histology, and Pharmacology, Faculty of Medicine, Universitas Airlangga, Jl. Prof. DR. Moestopo No.47, Pacar Kembang, Kec. Tambaksari, Surabaya, East Java 60132, Indonesia; h Division of Vascular and Endovascular Surgery, Department of Clinical Surgical and Health Sciences, University of Trieste, Strada di Fiume, 447 34149 Cattinara, Trieste, Italy

**Keywords:** Ascending aortic dilatation, Complication, Mortality, Safety, Transcatheter aortic valve implantation, Cardiovascular disease

## Abstract

The impact of ascending aorta dilatation (AAD) on transcatheter aortic valve implantation (TAVI) outcomes, compared to non-AAD, remains unclear. This *meta*-analysis aims to compare the outcomes of TAVI between patients with and without AAD. We systematically searched PubMed, ScienceDirect, Web of Science, Springer, Cochrane, and Clinicaltrials.gov. for articles up to 25 March 2024 (PROSPERO ID CRD42024526311). A total of 204,078 patients from ten studies were included. Paravalvular regurgitation (RR 1.56 95 %CI: 1.32–1.84, p < 0.00001, I^2^ = 0 %) and aortic dissection (RR 3.55 95 %CI: 1.79–7.06, p = 0.0003, I^2^ = 40 %) were more common in AAD group. However, there were no differences in *peri*-procedural (RR 1.09, 95 %CI: 0.83–1.42, p = 0.53, I^2^ = 0 %) and 1-year (RR 0.79, 95 %CI: 0.51–1.23, p = 0.30, I^2^ = 0 %) mortality. Three-years (RR 0.88, 95 %CI: 0.54–1.44, p = 0.62) and five-years (RR 0.85, 95 %CI: 0.45–1.6, p = 0.61) follow-up showed comparable mortality between both groups. The other complications and the need for second valve implantation (RR 1.24, 95 %CI: 0.70–20.20, p = 0.48, I^2^ = 65 %) were similar between both groups. Despite the higher incidence of aortic dissection and paravalvular regurgitation in AAD than in non-AAD patients, these complications were not associated with worse short-term or long-term mortality. Therefore, TAVI remains a safe and effective option for AAD patients.

## 1 Introduction

Aortic stenosis (AS) is the most prevalent valvular heart disease, affecting up to 12.4 % of the global population, particularly the elderly [1]. AS may be complicated by dilatation of the ascending aorta, with a prevalence reaching up to 25 % [2,3]. The European Society of Cardiology (ESC) and European Association for Cardio-Thoracic Surgery (EACTS) define pathologically dilated ascending aorta as ≥ 40 mm [4,5]. Patients with ascending aortic diameter ≥ 40 mm had 89 times higher risk of mortality compared to the normal population. In addition, higher diameter of ascending aorta is associated with higher mortality, such as less prevalent cases of aortic ascending aneurysms when ascending aortic diameter is ≥ 45 mm [6]. Yet, clinical implications are debated due to imaging variations and differing views on adjustments for age, gender, and body size [7].

American College of Cardiology (ACC) guidelines recommend simultaneous surgical aortic valve replacement (SAVR) and aortic tissue repair if ascending aorta diameter exceeds 45 mm to prevent acute type A ascending aortic dissection or rupture [8]. However, this is not feasible for patients with high-risk AS. With the increasing number of patients with ascending aorta dilatation (AAD) who are being considered for the procedure, the precise influence of AAD on transcatheter aortic valve implantation (TAVI) outcomes remains unclear. Recent observational studies suggest AAD may not have a significant effect, but these data are limited [9,10]. Therefore, this study aims to evaluate AAD's influence on TAVI outcomes.

## 2 Methods

### 2.1 Study design

This *meta*-analysis followed the 2020 Preferred Reporting Items for Systematic Review and Meta-analysis (PRISMA) guidelines (**Supplementary Table 1**) and was registered in the PROSPERO database (CRD42024526311) [11].

### 2.2 Eligibility criteria

This *meta*-analysis included observational cohort studies and randomized clinical trials. Case reports and case series were excluded from this review. The screening process involved evaluating titles and abstracts of retrieved studies based on the following eligibility criteria: (1) studies involving adult patients > 18 years of age who underwent TAVI; (2) comparing between AAD and non-AAD group; (3) reported at least one of our outcomes of interest; and (4) written in English. The primary outcome of this study is mortality, whereas the secondary outcomes include various procedural outcomes such as post-procedural left ventricular ejection fraction (LVEF) and complications, including paravalvular regurgitation (PVR), myocardial infarction, permanent pacemaker placement, aortic dissection, conversion to open surgery, and second valve implantation. There was no limitation on publication year. Studies with inaccessible full text, non-human subjects, reviews, and editorials were excluded. If the study fulfilled inclusion criteria but the reported data or full text was not available, we emailed the corresponding author.

### 2.3 Search strategy and study selection

Three authors performed a systematic literature search for studies published until 25 March 2024 in trial registries (ClinicalTrials.gov and the World Health Organization (WHO) Clinical Trial Registry) and medical databases (PubMed, Science Direct, Web of Science, Scopus, Springer, Cochrane). The following keywords were used: “(((((transcatheter aortic valve replacement) OR (TAVR)) OR (transcatheter aortic valve implantation)) OR (TAVI)) AND (Ascending Aorta Dilatation)) OR (AAD)”. Additionally, manual and bibliographical searches were conducted to obtain additional evidence and cover grey literature. The full texts of potentially eligible studies were subsequently screened independently by the same authors for inclusion in the final *meta*-analysis. If there were any disagreements, consensus between the authors was used to resolve the issue.

### 2.4 Data extraction and risk of bias assessment

Relevant data were extracted independently by two authors. Data that were extracted from included studies including study design, number of participants, number of males, age, body mass index (BMI), New York Heart Association (NYHA) functional class, Society of Thoracic Surgeons (STS) score, comorbidities (including smoking, hypertension, diabetes mellitus, dyslipidemia, coronary artery disease, atrial fibrillation, peripheral arterial disease, cerebrovascular disease), condition of aortic valve including bicuspid aortic valve & aortic valve, type of TAVI valve, echocardiographic parameters (including left ventricular ejection fraction (LVEF), transaortic gradient, aortic valve area, ascending aorta (AA) diameter), AA diameter based on CT scan measurements, follow-up duration, *peri*-procedural mortality, 1-year mortality, and post-procedural complications (including paravalvular regurgitation, aortic dissection, myocardial infarction, major vascular complications, permanent pacemaker placement (PPM), conversion to open surgery and second valve implantation). Any discrepancy was resolved by consensus. Peri-procedural mortality was defined as any mortality that occurred either during hospitalization or within 30 days following the procedure.

Two authors independently assessed the risk of bias in each study using the Newcastle-Ottawa Scales (NOS) (11). NOS categorized studies into three groups. Studies with ≥ 7 points were considered “good,” those with 2 to 6 points were considered “fair,” and those with ≤ 1 point were considered “poor” quality of study [12]. Any discrepancy was resolved by consulting with a third author. The result of the critical appraisal showed that none of the included studies had a high risk of bias **(Supplementary Table 2)**.

### 2.5 Statistical analysis

The impact of AAD on TAVI outcomes was measured using relative risk (RR) for binary outcomes and mean difference (MD) for continuous outcomes. Any continuous outcomes reported in the median and interquartile range were converted to MD with mean variance estimation formula [13,14]. The heterogeneity of the studies was assessed using *I^2^* statistics. When heterogeneity was considered high, the DerSimonian–Laird random-effects model was utilized. Sensitivity analysis was performed using the leave-one-out method. When possible, subgroup analyses were performed to explain heterogeneity and provide important clinical context. All statistical analyses were performed using Review Manager (version 5.4.1), and statistical significance was set at P < 0.05.

## 3 Results

### 3.1 Overview of included studies

**Fig. 1:**
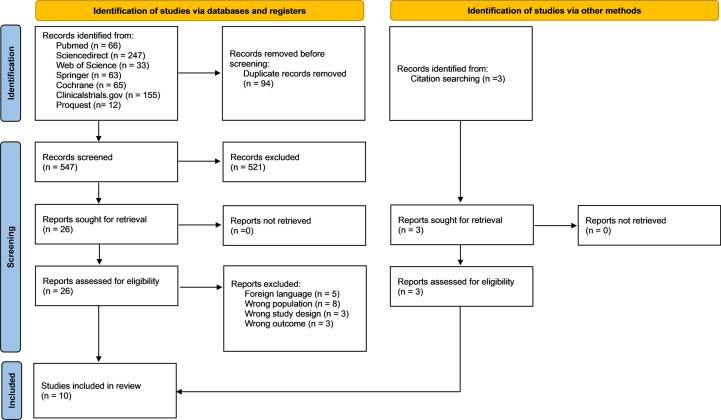
PRISMA flowchart of article selection.

A total of 641 articles were obtained and screened based on inclusion criteria. Then, 94 studies were excluded due to duplication. After thorough abstract screening and full-text review, ten studies [[15], [16], [17], [18], [19], [20], [21], [22], [23], [24]] were included in the *meta*-analysis. The PRISMA flow diagram **(**Fig. 1**)** outlines the selection process. An et al., (2023b) [15] was excluded due to population overlap with An et al., (2023a) [25], resulting in the inclusion of the latter study due to its larger sample size. The full text of a study by Ancona et al., (2019) [26] was irretrievable. Characteristics of included studies are summarized in Table 1**.**

**Table 1:** Baseline Characteristics of Included Studies.

**No.**	**Author, Year**	**Study Design**	**Number of Participants**		**Number of Male**		**Age**		**BMI**		**NYHA Class**		**STS score**	
			**AAD**	**Non-AAD**	**AAD**	**Non-AAD**	**AAD**	**Non-AAD**	**AAD**	**Non-AAD**	**AAD**	**Non-AAD**	**AAD**	**Non-AAD**
1	An et al., 2023 (14)	Retrospective Cohort	100	367	63 (63.0 %)	207 (56.4 %)	73 (69–77)	75 (70–80)	23.7 (21.1–26.7)	24.1 (22.1–27.2)	**NYHA Class III-IV** 89 (89.0 %)	**NYHA Class III-IV** 327 (89.1 %)	3.9 (3.4–5.0)	4.1 (3.7–5.1)
2	Boxhammer et al., 2023 (15)	Retrospective Cohort	32	32	24 (75 %)	24 (75 %)	82.06 ± 5.01	81.97 ± 5.41	27.12 ± 4.00	27.34 ± 3.84	3.00 ± 1.00	3.00 ± 0.75	2.26 ± 1.09	2.23 ± 1.61
3	Fan et al., 2024	Retrospective Cohort	49	62	12 (24.4 %)	26 (41.9 %)	73.9 ± 7.18	75.3 ± 7.2	23.4 ± 3.0	23.0 ± 3.1	**NYHA Class III-IV** 35 (71.4 %)	**NYHA Class III-IV** 46 (74.1 %)	N/A	N/A
4	Feng et al., 2024 (16)	Retrospective Cohort	107	449	72 (67.3 %)	251 (55.9 %)	73.9 ± 6.6	75.9 ± 7.4	23.6 ± 3.3	23.6 ± 3.6	**NYHA Class I**: 5 (4.7 %) **NYHA Class II:** 31 (29.0 %) **NYHA Class III**: 59 (55.1 %) **NYHA Class IV**: 12 (11.2 %)	**NYHA Class I**: 10 (2.2 %) **NYHA Class II**: 93 (20.7 %) **NYHA Class III**: 255 (56.8 %) **NYHA Class IV**: 91 (20.3 %)	N/A	
5	Kassis et al., 2018	Retrospective cohort	1677	169.334	1018 (66.1 %)	90.311 (53.3 %)	82 (71–86)	82 (76–87)	N/A	N/A	N/A	N/A	N/A	N/A
6	Kobayashi et al., 2018	Retrospective Cohort	22	210	10(45.5 %)	115 (54.8 %)	83 (71–88)	83 (75–86)	26 (25–27)	27 (24–30)	N/A	N/A	7.9 (6.1–12.3)	6.6 (4.6–10.1)
7	Ochiai et al., 2020 (17)	Prospective Cohort	196	1230	N/A		N/A		N/A		N/A		N/A	
8	Rylski et al., 2014 (18)	Retrospective Cohort	98	357	70 (71.4 %)	158 (44.3 %)	85.0 (9.0)	85.2 (8.6)	N/A		**NYHA Class I**:0 (0 %) **NYHA Class II:** 5 (5.1 %) **NYHA Class III**: 51 (52.0 %) **NYHA Class IV:** 42 (42.9 %)	**NYHA Class I**: 3 (0.8 %) **NYHA Class II:** 24 (6.7 %) **NYHA Class III:** 192 (53.8 %) **NYHA Class IV**: 133 (37.3 %)	**High risk** 10 (4.8 %)	**High risk**11 (4.5 %)
9	Ugwu et al., 2024	Retrospective Cohort	910	28.607	551 (71.2 %)	565 (73.0 %)	80.1 ± 8.7	80.4 ± 8.1	N/A	N/A	N/A	N/A	N/A	N/A
10	Yu & Wang, 2023 (19)	Retrospective Cohort	73	166	207 (56.4 %)	86 (51.8 %)	73.7 ± 7.3	73.1 ± 7.3	24.2 ± 3.1	25.2 ± 3.8	**NYHA Class III-IV** 52 (71.2 %)	**NYHA Class III-IV** 115 (69.3 %)	6.0 (5.0–7.0)	6.0 (5.0–7.0)

### 3.2 Baseline and clinical characteristics of study subjects

The review included 204,078 patients, predominantly male (up to 75 %), with a mean age ranging from 73.1 to 85.2 years old. The average BMI ranged from 23.6 to 27.34 kg/m^2^, falling between the normal and overweight categories according to the WHO [27]. Included participants predominantly classified as NYHA Class III-IV [28]. To stratify the procedural risk of patients undergoing aortic valve replacement (AVR), most studies collected STS scores ranging from 2.23 to 7.9, which can be classified as moderate to high risk [29]. Moreover, studies by An et al., (2023) [15], Fan et al., 2024 [17], Feng et al., (2024) [18], Kassis et al., (2018) [19], Kobayashi et al., 2018 [20] and Yu & Wang (2023) [24] also documented additional conditions of aortic valve including bicuspid aortic valve (BAV). Further details regarding the comorbidities of included samples were summarized in **Supplementary Table 3**.

### 3.3 Procedural details

Eight included studies [[15], [16], [17], [18],[20], [21], [22],^24^] documented pre-procedural LVEF ranging from 43.5 % to 65 % and could be classified between mildly reduced and normal [30]. Post-procedural LVEF data was available in only two studies by An et al., (2023) [15] and Yu & Wang (2023) [24], showing lower value in the AAD group ranging from 55.9 to 58.2 %. In addition, most included studies collected [[15], [16], [17], [18],[20], [21], [22],^24^] pre-procedural transaortic gradients, ranging from 34 to 108.2 mmHg, which could be classified between normal to elevated range [31]. Two imaging modalities were used to measure AA diameter: echocardiography and CT scan. Among the three [15,21,22] studies using echocardiography, AA diameter ranged from 31 to 46 mm, while four studies using CT scans reported a range of 34.99 to 50.6 mm [16,18,19,24]. A study by Feng et al., (2024) also particularly documented initial and post-procedural AA diameter at the follow-up period (median 3.9 years; 95 % CI:3.8–4.0 years) and revealed no significant changes (increase or decrease ≥ 2 mm) of AA diameter, regardless of BAVs and TAVs grouping. Unfortunately, other studies did not provide detailed data regarding the outcome differences between BAV and TAV. Details regarding procedural details are presented in [18] Table 2.

**Table 2:** Peri- and Post-procedural Characteristics.

**No**	**Author, Year**	**Echocardiographic Parameter**								**Ascending aorta diameter based on CT scan**		**Follow up duration**		**Type of TAVI Valve**		**Mortality**	
		**LVEF (%)**		**Transaortic gradient (mmHg)**		**Aortic valve area (cm2)**		**Ascending aorta diameter (mm)**									
		**AAD**	**Non-AAD**	**AAD**	**Non-AAD**	**AAD**	**Non-AAD**	**AAD**	**Non-AAD**	**AAD**	**Non-AAD**	**AAD**	**Non-AAD**	**AAD**	**Non-AAD**	**AAD**	**Non-AAD**
1	An et al., 2023	**Initial:** 60 (43.5–65) **Post-Procedural:** 60 (47.3–65)	**Initial:** 60 (51–65) **Post-Procedural:** 60 (55–65)	**Initial:** 88.4 (74.0–108.2) **Post-Procedural:** 21.2 (17.6–30.9)	**Initial:** 88.4 (74.0–108.2) **Post-Procedural:** 23.0 (16.0–29.2)	N/A	N/A	**Initial:** 45 42–48) **Follow-up:** 46 (44–48)	**Initial:** 35 (32–38) **Follow-up:** 35 (31–38)	N/A	N/A	19 (16–34) months	27 (15–37) months	**BEV:** 9 (9.0 %) **SEV:** 91 (91.0 %)	**BEV:** 53 (14.4 %) **SEV:** 314 (85.6 %)	**In-hospital Mortality:** 0 (0 %) **All-Cause Mortality:** 0 (0.0 %)	**In-hospital Mortality:** 5 (1.4 %) **All-Cause Mortality:** 5 (1.4 %)
2	Boxhammer et al., 2023	**Initial:** 52.41 ± 11.53	**Initial:** 56.63 ± 12.15	46.44 ± 10.01	49.39 ± 13.93	N/A	N/A	N/A	N/A	**Initial:** 42.24 ± 2.19	**Initial:** 34.99 ± 2.48	N/A	N/A	N/A	N/A	**1-year Mortality:** 10 (31.2 %) **3-year Mortality:** 15 (45.2 %) **5-year Mortality:** 11 (36.0 %)	**1-year Mortality:** 12 (37.5 %) **3-year Mortality:** 17 (54.8 %) **5-year Mortality:** 13 (40.0 %)
3	Fan et al., 2024	**Initial:** 55.6 ± 12.0	**Initial:** 56.0 ± 12.9	**Initial:** 59.2 ± 28.5	**Initial:** 90.1 ± 29.0	**Initial:** 517.3 ± 108.5	**Initial:** 472 ± 11.6	N/A		**Initial:** 43.3 ± 2.8	**Initial:** 35.9 ± 3.8	54.1 ± 23.9 months	59.3 ± 17. months	All patients used SEV		**In-hospital mortality:** 2 (4.2 %) **All-cause mortality:** 14 (28.5 %) **Cardiovascular mortality:** 5 (10.2 %)	**In-hospital mortality:** 1 (1.6 %) **All-cause mortality:** 7 (11.2 %) **Cardiovascular mortality:** 3 (4.8 %)
4	Feng et al., 2024	**Initial:** 53.9 ± 14.5	**Initial:** 55.6 ± 13.3	**Initial:** 57.6 ± 19.5	**Initial:** 55.8 ± 17.1	N/A	N/A	N/A	N/A	**Initial:** 47.8 (46.5–50.4) **Follow-up:** 47.9 (46.2–50.6)	**Initial:** 38.0 (34.7–40.5) **Follow-up:** 37.8 (35.2–40.9)	1.9 (1.1–––2.7) years	1.3 (1.0–2.2) years	**BEV:** 100 (93.5 %) **SEV:** 7 (6.5 %)	**BEV:** 409 (92.5 %) **SEV:** 33 (7.5 %)	**In-hospital mortality:** 3 (2.8 %) **All-cause mortality:** 9 (8.9 %) **4-year Mortality:** 5 (5.1 %)	**In-hospital mortality:** 5 (1.1 %) **All-cause mortality:** 89 (19.9 %) **4-year Mortality:** 39 (8.7 %)
5	Kassis et al., 2018	N/A	N/A	N/A	N/A	N/A	N/A	N/A	N/A	N/A	N/A	N/A	N/A	156 (9.3 %)	1,450 (0.9 %)	**In-hospital mortality:** 43 (2.6 %)	**In-hospital mortality:** 4.217 (2.5 %)
6	Kobayashi et al., 2018	**Initial:** 55 (50–60)	**Initial:** 55 (45–60)	**Initial:** 40 (37–45)	**Initial:** 41 (34–51)	N/A	N/A	**Major annulus:** 26 (23–27) **Minor annulus:** 21 (19–22)	**Major annulus:** 26 (24–29) **Minor annulus:** 22 (20–23)	N/A	N/A			3 (13.6 %)	9 (4.3 %)	**In-hospital mortality:** 1 (4.5 %) **6-months mortality:** 2 (9.1 %)	**In-hospital mortality:** 12 (5.7 %) **6-months mortality:** 16 (7.6 %)
7	Ochiai et al., 2020	**Initial:** 54.4 ± 15.3	**Initial:** 57.8 ± 14.5	41.3 ± 13.8	43.5 ± 13.5	54.0 (42.0–67.0)	50.0 (41.0–63.3)	N/A	N/A	N/A	N/A	391 (99 to 727) days.		N/A	N/A	**30-days mortality:** Not significant, but no data was presented **2-year all-cause mortality:** 70 (34.5 %)	**30-days mortality:** Not significant, but no data was presented **2-year all-cause mortality:** 251 (20.4 %)
8	Rylski et al., 2014	**Initial:** 60 ± 25	**Initial:** 60 ± 20	46 ± 15	46 ± 16	0.7 ± 0.2	0.5 ± 0.2	4.1 ± 0.2	3.1 0.5	N/A	N/A	14 months		N/A	N/A	**In-hospital mortality:** 7 (7.1 %) **1-year mortality:** 10 (13.3 %)	**In-hospital mortality:** 17 (4.8 %) **1-year mortality:** 41 (16.9 %)
9	Ugwu et al., 2024	N/A	N/A	N/A	N/A	N/A	N/A	N/A	N/A	N/A	N/A	**In-hospital** AAD: 4 (2.0–7.0) days Non-AAD: 3 (2.0–6.0) days		N/A	N/A	N/A	**In-hospital mortality:** 16 (2.0 %)
10	Yu & Wang, 2023	**Post-** **Procedural:** 60.0 (46.5–65.0)	**Post-** **Procedural:** 60.0 (50.0–65.0)	54.0 (42.0–67.0)	50.0 (41.0–63.3)	N/A	N/A	44.0 (42.0–46.0)	36.0 (34.0–37.0)	44.0 (42.0–46.0)	36.0 (34.0–37.0)	588 (384–1014) days		**BEV:** 8 (11 %) **SEV:** 65 (89 %) **Balloon predilatation:** 63 (86.3 %) **Balloon post-dilatation:** 17 (23.3 %)	**BEV:** 19 (11.4 %) **SEV:** 147 (88.6 %) **Balloon predilatation:** 149 (89.8 %) **Balloon post-dilatation:** 20 (12 %)	*All-cause mortality* ***Periprocedural (30 days)*:** 1 (1.4 %) ***Early (1 year)*:** 3 (5.0 %) ***Late (3 years)*:** 4 (9.5 %) *Cardiovascular mortality* ***Periprocedural (30 days)*:** 0 (0.0 %) ***Early (1 year)*:** 1 (2.0 %) ***Late (3 years)*:** 1 (1.9 %) *Valve related mortality* ***Periprocedural (30 days)*:** 0 (0.0 %) ***Early (1 year)*:** 0 (0.0 %) ***Late (3 years)*:** 0 (0.0 %)	*All-cause mortality* ***Periprocedural (30 days)*:** 4 (2.0 %) ***Early (1 year)*:** 15 (9.2 %) ***Late (3 years)*:** 18 (13.1 %) *Cardiovascular mortality* ***Periprocedural (30 days)*:** 3 (1.8 %) ***Early (1 year)*:** 12 (7.7 %) ***Late (3 years)*:** 13 (10.0 %) *Valve related mortality* ***Periprocedural (30 days)*:** 1 (0.6 %) ***Early (1 year)*:** 3 (1.9 %) ***Late (3 years)*:** 3 (1.9 %)

### 3.4 Mortality assessment in individual studies

**Fig. 2:**
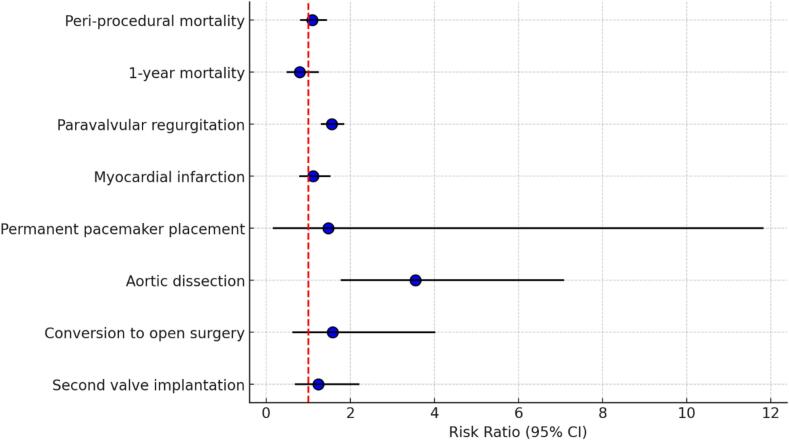
The summary of TAVI outcomes in patients with AAD.

Mortality assessment differed across included studies by An et al., (2023) [15], Feng et al., (2024) [18], and Yu & Wang (2023) [24] documented that all-cause mortality was more common in the non-AAD patients (AAD vs non-AAD: 0.0 % vs 1.4 %; 8.9 % vs 10.9 %; 10.9 % vs 22.3 %). Included studies, particularly Boxhammer et al., 2023 [16] also reported that the non-AAD patients had higher mortality rates at 1-year, 3-year, and 5-year intervals following the procedure, compared to the patients with AAD (1-year mortality: 31.2 % vs 37.5 %; 3-year mortality: 45.2 % vs 54.8 %; 5-year mortality: 36.0 % vs 40.0 %). In contrast, Ochiai et al., (2020) [21] reported a higher 2-year mortality rate in the AAD group (AAD vs Non-AAD: 34.5 % vs 20.4 %). Yu & Wang, 2023 [24] documented that the mortality rate remained consistently higher in the non-AAD patients at 30 days, 1-year, and 3-year time points following the procedure, compared to the patients with AAD (30-days mortality; 1.4 % vs 4.8 %; 1-year mortality: 5.5 % vs 18.1 %; 3-year mortality: 5.5 % vs 20.5 %). In addition, Feng et al., (2024) [18] also reported that subgroup analysis between BAV and TAV revealed no significant difference in all-cause and cardiovascular mortality at 1-year and even at 4-year time points (see Fig. 2.).

### 3.5 Pooled peri-procedural mortality

Seven studies [15,[17], [18], [19], [20],^22^,^24^] with a total of 173,071 patients documented *peri*-procedural mortality. Following a random-effects analysis, the pooled outcomes of *peri*-procedural mortality did not reveal significant differences, with negligible heterogeneity (RR 1.09 95 %CI: 0.83, 1.42, p = 0.53, I^2^ = 0 %). Due to the negligible heterogeneity, subgroup analysis was not performed (Fig. 1**; Supplementary Fig. 5a**).

### 3.6 Pooled 1-year mortality

For 1-year mortality, three included studies [16,22,24] involving 758 patients were analyzed. The pooled analysis using a random-effects model showed no significant difference between AAD and non-AAD groups, with low heterogeneity (RR 0.79 95 %CI: 0.51, 1.23, p = 0.30, I^2^ = 0 %) (Fig. 1**; Supplementary Fig. 5b**).

### 3.7 Post-procedural complications (**Supplementary Table 6**)

#### 3.7.1 Paravalvular regurgitation

Four studies [15,18,22,24] with a total of 2688 patients were included in the analysis. In a study by An et al., (2023) [15] classified their samples into none or mild and moderate-severe PVR. Hence, only participants with moderate-severe PVR were included in this study [15]. Similarly, Feng et al., (2024) [18] only documented moderate-severe PVR [18]. The pooled analysis using a random-effects model showed that the AAD group had a significantly higher risk of post-procedural PVR compared to the non-AAD group, with a 56 % increased risk (RR 1.56 95 %CI: 1.32, 1.84, p < 0.00001, I^2^ = 0 %) (Table 1). Subgroup analysis was not performed due to low heterogeneity (Fig. 1**; Supplementary Fig. 7**).

#### 3.7.2 Myocardial infarction

Five studies [15,[17], [18], [19],^24^] with a total of 172.384 patients were included in the analysis. The outcomes were combined using a random-effects model and revealed no significant difference with minimal heterogeneity (RR 1.11 95 %CI: 0.81, 1.51, p = 0.51, I^2^ = 0 %) (Fig. 1**; Supplementary Fig. 8**).

#### 3.7.3 Permanent-pacemaker placement

Six studies [15,17,18,20,23,24] with a total of 172.384 patients were included in the analysis. The pooled results using a random-effects analysis showed no significant difference with high heterogeneity between the AAD and Non-AAD groups (RR 1.48 95 %CI: 0.28, 11.80, p = 0.72, I^2^ = 98 %) (Fig. 1**; Supplementary Fig. 9**). Leave-one-out analysis revealed similar heterogeneity after omitting each individual.

#### 3.7.4 Aortic dissection

Four studies [15,17,19,24] with a total of 172.145 patients were included in the analysis. The follow-up period ranged from 19 to 54.1 months in the AAD group and 15.6 to 59.3 months in the non-AAD group. Studies by An et al., (2023) [15] and Fan et al., 2024 [17] reported no case of post-procedural aortic dissection in the AAD group [15,17]. Another study by Yu & Wang (2023) [24] even reported no case of post-procedural aortic dissection in both AAD and non-AAD group [24]. The pooled analysis of post-procedural aortic dissection using a random-effects model demonstrated a significantly higher risk in the AAD group compared to the non-AAD group, with a 3.55-fold increased risk (RR 3.55 95 %CI: I.79, 7.06, p = 0.0003, I^2^ = 40 %), with moderate heterogeneity (Fig. 1**; Supplementary Fig. 10**).

#### 3.7.5 Conversion to open surgery

Three studies [15,18,24] with 1262 patients collected the prevalence of conversion to open surgery during the procedure. The pooled outcome of conversion to open surgery with a random effects model did not yield statistical significance with moderate heterogeneity between the AAD and Non-AAD patients (OR 1.58 95 %CI: 0.62, 4.00, p = 0.34, I^2^ = 60 %) (Fig. 1**; Supplementary Fig. 11**).

#### 3.7.6 Second valve implantation

Four studies [15,18,22,24] with a total of 2688 patients were included to assess post-procedural second valve implantation. The combined outcome of second valve implantation using a random effects model showed a non-significant difference with moderate heterogeneity between the AAD and Non-AAD patients (OR 1.24 95 %CI: 0.70, 2.20p = 0.48, I^2^ = 65 %) (Fig. 1**; Supplementary Fig. 12**).

## 4 Discussion

This study, encompassing more than 200.000 patients, aimed to provide contemporary systematic evidence regarding the impact of AAD on TAVI outcomes, including various aspects of mortality and complications. The included participants were dominated by males aged above 70 years old. Most participants were also classified as NYHA Class III-IV, with moderate to high-risk STS scores reflecting that the participants of these studies were according to the TAVI preference in various guidelines [28,29].

In terms of mortality, there were no significant differences between AAD and non-AAD patients. The periprocedural mortality was similar between both groups. That finding was consistent even during 1-year, 3-year, and 5-year follow-up. This shows that AAD was not associated with short-term or long-term mortality. The pooled analysis also confirmed that mortality after TAVI was independent of aortic events, reinforcing the hypothesis that AAD does not directly contribute to adverse survival outcomes. Additionally, a previous study of Mckellar et al., (2010) [32] also showed that aortic dilatation was not accountable for mortality following aortic valve replacement [32].

This *meta*-analysis revealed that the event of aortic dissection was higher in AAD compared to non-AAD patients. However, the absolute incidence remained low, suggesting that while AAD increases the relative risk, the overall clinical impact may be limited. As a note, only a study conducted by Kassis et al., (2018) [19] showed that AAD was significantly associated with aortic dissections compared to non-AAD patients in *meta*-analysis. The other risk factors for aortic dissection, such as older age, female sex, atherosclerosis, congestive heart failure, and diabetes mellitus, were more prevalent in AAD patients and might act as confounding factors [19]. Although the incidence of aortic dissection was significantly higher in the patients with AAD, its absolute value was remarkably low (only 1 %) [19]. Various factors may trigger post-procedural aortic dissection, one of which is the utilization of self-expandable valves (SEV) that can complicate the procedure and increase the risk of injury to the ascending aorta [9]. The other potential risk factor was the use of a bicuspid aortic valve (BAV), as it is associated with structural fragility and higher hemodynamic burden [37]. However, a study by Fan et al., (2024) [17] that exclusively examined BAV patients reported no post-procedural aortic dissections in the AAD group, suggesting that additional factors may modulate risk [17]. Despite these variations, the association between AAD and increased aortic dissection remained significant even after adjusting for confounders and performing subgroup analyses [19].

This *meta*-analysis also showed that paravalvular regurgitation (PVR) was more prevalent in AAD compared to non-AAD patients. This finding is clinically relevant as PVR has been linked to an increased risk of long-term mortality and late bleeding events. The proposed underlying mechanism involves the loss of high-molecular-weight von Willebrand factor due to the high shear stress and flow turbulence caused by PVR [34]. This issue can be managed through meticulous enhancement of technique, primarily by using 3D computed tomography for valve sizing and selection, and advancements in technology, such as repositionable valves and the addition of sealing skirts, which have proven to significantly reduce the rate of post-TAVI PVR [35]. The presence of a BAV and the use of SEV are also key factors that influence the risk of PVR. BAV is associated with elliptical annuli, asymmetric leaflet distribution, and increased calcification, which may lead to suboptimal prosthesis sealing and higher PVR rates compared to tricuspid aortic valves (TAV). Similarly, SEVs, due to their radial expansion mechanism, have been linked to higher PVR rates than balloon-expandable valves (BEVs), particularly in patients with heavily calcified or non-circular annuli [36,37]. However, newer SEV designs with enhanced sealing skirts and repositionable frames have improved outcomes. Future studies should further stratify PVR risk based on valve type and morphology in order to optimize procedural strategies.

Although the *meta*-analysis showed a higher incidence of aortic dissection and PVR in AAD compared to non-AAD, these complications did not lead to an increased need for conversion to surgery or second valve implantation. Additionally, there were also no significant differences in *peri*-procedural mortality or higher 1-year mortality rates between groups. However, given the retrospective nature of the included studies, these findings should be interpreted cautiously, as unmeasured confounders and potential biases may influence outcomes. Other complications such as myocardial infarction, stroke, the need for blood transfusion, and permanent pacemaker implantation were also similar between AAD and non-AAD groups [15,17,18,20,23,24]. It is also important to note that the study population was generally elderly and high-risk, which could influence the overall rates of vascular complications in AAD patients. While the primary endpoints were comparable, the other important parameter is quality of life. However, none of the included studies evaluate the quality of life between AAD and non-AAD patients. Although post-procedural LVEF was lower in AAD patients, the difference was not clinically significant and did not appear to impact overall prognosis [38].

One of the important considerations in AS treatment in the setting of AAD is dynamic changes in aortic diameter. As dilated aortic diameter changes gradually during follow-up time, the need to use TAVI instead of SAVR in AS with AAD should be considered. To this date, studies focusing on changes in the AA diameter in the AAD population following TAVI were scarce. A study conducted by An et al., (2023a) [15] specifically observed ascending aortic diameter pre- and post-TAVI using echocardiography, which revealed no significant changes in diameter. Another study by Feng et al., (2024) [18] used CT scan measurements and also concluded that no significant changes in AA diameter were observed in both bicuspid and tricuspid aortic valves [18]. Surprisingly, a study by Lv et al., (2019) [10] revealed a slight decrease in AA diameter following TAVI in patients with mild aortic dilation [10]. This condition is assumed to be caused by the correction of hemodynamic disturbances due to valve dysfunction. Similarly, He et al., (2019) also documented minimal non-significant progression of AAD after TAVI [9]. These findings suggest that TAVI does not accelerate aortic enlargement in AAD patients and that concerns regarding post-procedural aortic remodeling should not preclude its use [22,33,[39], [40], [41]].

According to the 2021 ESC and EACTS guidelines, SAVR is indicated, an aortic diameter of 45 mm or more suggests concomitant repair of the aortic root or tubular ascending aorta [5]. While endovascular repair is effective in treating descending aortic pathologies, its application for ascending aortic pathologies remains limited to highly selected cases owing to several anatomical constraints in this area [42,43]. Consequently, SAVR remains to be the preferred method when treating patients with AAD [44]. The decision between SAVR and TAVI should consider various factors, including the team's experience, the presence of aortic root aneurysm, cusp characteristics, life expectancy, and desired anticoagulation status [6,45,46]. The findings of this *meta*-analysis support TAVI as a viable alternative in select AAD patients, particularly those deemed unsuitable for SAVR. A more conservative approach for treating AAD can also be considered, supported by evidence of modest procedural-related complications. With the development of newer technology and non-invasive methods, further evidence related to the long-term outcomes and progression of aortic diameters after TAVI should be gathered to support any shifts in clinical practice.

## 5 Strengths and limitations

This *meta*-analysis presents several strengths and limitations. First, most follow-up data extended only up to 1 year, with limited studies reporting beyond 3 years and none exceeding 5 years, limiting the generalizability of long-term outcomes. Second, due to reliance on secondary data, detailed anatomical characteristics of aortic dilation, such as PVR location, were unavailable, which may have influenced outcomes. Third, heterogeneity was high for variables such as the need for surgery and second valve implantation, but leave-one-out sensitivity analyses confirmed consistent results. In contrast, mortality outcomes demonstrated low heterogeneity, reinforcing the robustness of our findings. A sensitivity analysis for BAV vs. non-BAV was attempted, but stratified data were inconsistently reported across studies, preventing its completion. This represents a limitation, as bicuspid valve morphology may influence outcomes in AAD patients undergoing TAVI. Future studies should aim to provide subgroup data to refine risk assessment in this population.

This *meta*-analysis has several strengths. It is the largest study to date evaluating TAVI outcomes in AAD patients, including over 200,000 patients. The assessment of eight endpoints provides a comprehensive evaluation of safety and efficacy. Furthermore, most outcomes exhibited low heterogeneity, enhancing confidence in the pooled estimates. Additionally, this study adds valuable insights into post-TAVI changes in ascending aortic diameter, supporting the feasibility of TAVI in this population. In an era of evolving transcatheter therapies, these findings support TAVI as a viable option for AAD patients unsuitable for SAVR. Further research should focus on patient selection, procedural optimization, and long-term outcomes to guide future clinical practice.

## 6 Conclusions

Post-TAVI complications were generally comparable between AAD and non-AAD groups, except for a higher risk of aortic dissection and paravalvular regurgitation in the AAD group. However, the rates of conversion to surgery and second valve implantation did not differ significantly between groups. Periprocedural mortality was also similar, with consistent outcomes during both short-term and long-term follow-up. Given these findings, TAVI appears to offer a comparable safety and efficacy profile in AAD patients, though further research is warranted to address potential biases and long-term outcomes.

## 7 Informed consent statement

None

## 8 Key References

1. An K, Zhang F, Ouyang W, et al. Transcatheter aortic valve replacement in patients with preoperative ascending aortic diameter ≥45 mm. Cardiovasc Diagn Ther. 2023 Dec 15;13(6):939–47

This study is crucial because it investigates the outcomes of TAVI in patients with significant preoperative ascending aorta dilatation, providing critical data on the safety and efficacy of the procedure in this specific patient population.

2. Feng D, Zhao J, Niu G, et al. Outcomes for patients undergoing transcatheter aortic valve replacement with ascending aorta dilation. Int J Cardiol. 2024 Mar 11;131948.

This recent publication offers valuable insights into the peri- and post-procedural outcomes of TAVI in patients with ascending aorta dilation, contributing to the understanding of how aortic dilatation influences TAVI results and patient prognosis.

3. Yu J, Wang W. Short- to mid-term outcomes after transcatheter aortic valve replacement in patients with ascending aorta dilation: a single-centre retrospective analysis. BMC Cardiovasc Disord. 2023 Jan 18;23(1):31.

This study provides additional detailed analysis regarding the short- to mid-term outcomes post-TAVI in patients with ascending aorta dilation, highlighting the procedure's feasibility and safety in a real-world clinical setting

Funding.

The author(s) receive no specific grant from any funding.

## CRediT authorship contribution statement

**Pandit Bagus Tri Saputra:** Writing – review & editing, Writing – original draft, Visualization, Validation, Software, Resources, Project administration, Methodology, Investigation, Formal analysis, Data curation, Conceptualization. **Wynne Widiarti:** Writing – review & editing, Writing – original draft, Visualization, Software, Resources, Methodology, Investigation, Formal analysis, Data curation, Conceptualization. **Ali Mustofa:** Writing – review & editing, Writing – original draft, Supervision, Resources, Methodology, Investigation, Data curation, Conceptualization. **Cornelia Ghea Savitri:** Writing – review & editing, Writing – original draft, Software, Project administration, Methodology, Investigation, Data curation, Conceptualization. **Johanes Nugroho Eko Putranto:** Writing – review & editing, Writing – original draft, Visualization, Supervision, Software, Resources, Methodology, Investigation, Funding acquisition, Data curation, Conceptualization. **Faisal Yusuf Ashari:** Writing – review & editing, Writing – original draft, Validation, Supervision, Project administration, Methodology, Formal analysis. **Chaq El Chaq Zamzam Multazam:** Writing – review & editing, Writing – original draft, Visualization, Project administration, Investigation, Funding acquisition, Conceptualization. **Firas Farisi Alkaff:** Writing – review & editing, Writing – original draft, Visualization, Supervision, Software, Resources, Project administration, Investigation, Funding acquisition, Conceptualization. **Mario D’Oria:** Writing – review & editing, Writing – original draft, Supervision, Resources, Project administration, Funding acquisition, Formal analysis, Conceptualization.

## Declaration of competing interest

The authors declare that they have no known competing financial interests or personal relationships that could have appeared to influence the work reported in this paper.
