# Supplementary material for: The impact of ascending aorta dilatation on transcatheter aortic valve implantation outcomes
Source: Int J Cardiol Heart Vasc. 2025 Apr 19;58:101680. doi: 10.1016/j.ijcha.2025.101680 (PMC12023879; doi:10.1016/j.ijcha.2025.101680)
Supplement: Supplementary Data 1 [file mmc1.docx]

**Title:** The Impact of Ascending Aorta Dilatation on Transcatheter Aortic Valve Implantation (TAVI) Outcomes: Systematic Review and Meta Analysis

**Supplementary Table 1.** PRISMA Checklist

**Supplementary Table 2.** Risk of Bias Assessment of Included Observational Studies according to the Newcastle-Ottawa Assessment Scales

**Supplementary Table 3.** Baseline Characteristics of Included Studies

**Supplementary Table 4.** Comorbidities of Patients in Included Studies

**Supplementary Table 5.** Forrest Plot for Post-Procedural LVEF

**Supplementary Table 6.** Peri- and Post- Procedural Characteristics

**Supplementary Table 7.** Forrest Plot for Post-Procedural Mortality

**Supplementary Table 8.** Post-procedural Complications

**Supplementary Table 9.** Forrest Plot for Post-Procedural Paravalvular Regurgitation

**Supplementary Table 10.** Forrest Plot for Post-Procedural Myocardial Infarction

**Supplementary Table 11.** Forrest Plot for Post-Procedural Permanent-Pacemaker Placement

**Supplementary Table 12.** Forrest Plot for Post-Procedural Aortic Dissection

**Supplementary Table 13.** Forrest Plot for Post-Procedural Conversion to Open Surgery

**Supplementary Table 14.** Forrest Plot for Post-Procedural Second Valve Implantation

**Supplementary Table 1.** PRISMA Checklist


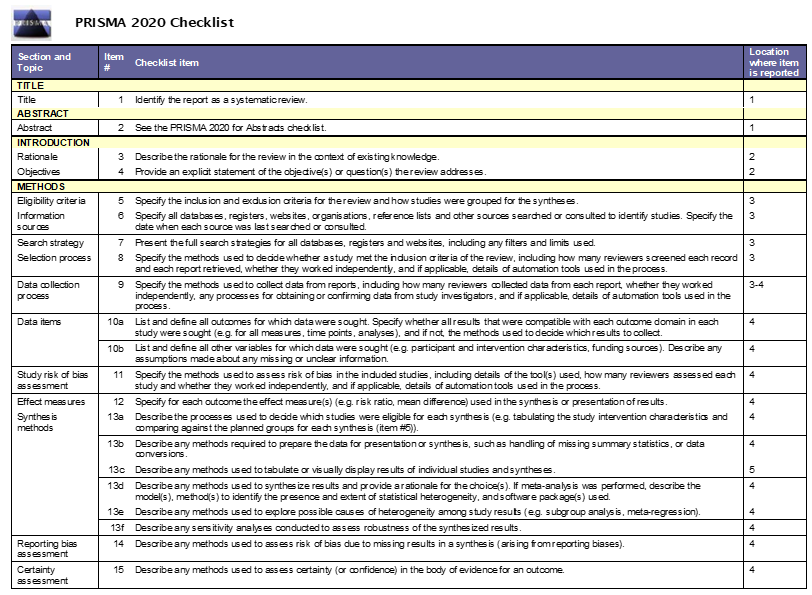


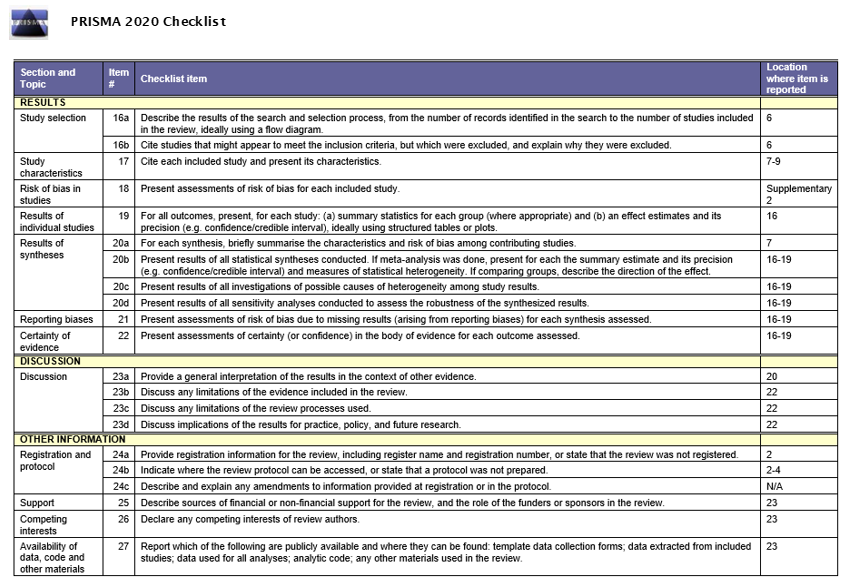


**Supplementary Table 2.** Risk of Bias Assessment of Included Observational Studies

| **Author, Year** | **Study** | **Selection** | **Comparability** | **Outcome** | **Total** | **Category** |
| --- | --- | --- | --- | --- | --- | --- |
| An *et al.*, 2023 | Cohort study | 4 | 2 | 3 | 9 | Low Risk |
| Boxhammer *et al.,* 2023 | Cohort study | 3 | 2 | 3 | 8 | Low Risk |
| Fan et al., 2024 | Cohort study | 3 | 2 | 3 | 8 | Low Risk |
| Feng *et al.,* 2024 | Cohort study | 4 | 2 | 3 | 9 | Low Risk |
| Kassis et al., 2018 | Cohort study | 2 | 2 | 2 | 6 | Moderate  Risk |
| Kobayashi et al., 2018 | Cohort study | 3 | 2 | 2 | 7 | Low Risk |
| Ochiai *et al.,* 2020 | Cohort study | 2 | 2 | 2 | 6 | Moderate Risk |
| Rylski *et al.,* 2014 | Cohort study | 4 | 2 | 3 | 9 | Low Risk |
| Ugwu et al., 2024 | Cohort study | 2 | 2 | 2 | 6 | Moderate  Risk |
| Yu & Wang *et al.,*2023 | Cohort study | 4 | 2 | 3 | 9 | Low Risk |

**Supplementary Table 3.** Baseline Characteristics of Included Studies

| **No.** | **Author, Year** | **Study Design** | **Number of Participants** | | **Number of Male** | | **Age** | | **BMI** | | **NYHA Class** | | **STS score** | |
| --- | --- | --- | --- | --- | --- | --- | --- | --- | --- | --- | --- | --- | --- | --- |
|  |  |  |  |  |  |  |  |  |  |  |  |  |  |  |
|  |  |  | **AAD** | **Non-**  **AAD** | **AAD** | **Non-**  **AAD** | **AAD** | **Non-**  **AAD** | **AAD** | **Non-**  **AAD** | **AAD** | **Non-**  **AAD** | **AAD** | **Non-**  **AAD** |
| 1 | An et al., 2023 [(14)](https://www.zotero.org/google-docs/?CwertH) | Retrospective Cohort | 100 | 367 | 63 (63.0%) | 207 (56.4%) | 73 (69–77) | 75 (70–80) | 23.7 (21.1–26.7) | 24.1 (22.1–27.2) | **NYHA Class III-IV**  89 (89.0%) | **NYHA Class III-IV**  327 (89.1%) | 3.9 (3.4–5.0) | 4.1 (3.7–5.1) |
|  |  |  |  |  |  |  |  |  |  |  |  |  |  |  |
| 2 | Boxhammer et al., 2023 [(15)](https://www.zotero.org/google-docs/?2txiBU) | Retrospective Cohort | 32 | 32 | 24 (75%) | 24 (75%) | 82.06 ± 5.01 | 81.97 ± 5.41 | 27.12 ± 4.00 | 27.34 ± 3.84 | 3.00 ± 1.00 | 3.00 ± 0.75 | 2.26 ± 1.09 | 2.23 ± 1.61 |
| 3 | Fan et al., 2024 | Retrospective Cohort | 49 | 62 | 12 (24.4%) | 26 (41.9%) | 73.9 ± 7.18 | 75.3 ± 7.2 | 23.4 ± 3.0 | 23.0 ± 3.1 | **NYHA Class III-IV**  35 (71.4%) | **NYHA Class III-IV**  46 (74.1%) | N/A | N/A |
| 4 | Feng et al., 2024 [(16)](https://www.zotero.org/google-docs/?wgBWcM) | Retrospective Cohort | 107 | 449 | 72 (67.3%) | 251 (55.9%) | 73.9 ± 6.6 | 75.9 ± 7.4 | 23.6 ± 3.3 | 23.6 ± 3.6 | **NYHA Class I**:  5 (4.7%)  **NYHA Class II:** 31 (29.0%)  **NYHA Class III**: 59 (55.1%)  **NYHA Class IV**: 12 (11.2%) | **NYHA Class I**: 10 (2.2%)  **NYHA Class II**: 93 (20.7%)  **NYHA Class III**: 255 (56.8%)  **NYHA Class IV**: 91 (20.3%) | N/A | |
| 5 | Kassis et al., 2018 | Retropsective cohort | 1677 | 169.334 | 1018 (66.1%) | 90.311 (53.3%) | 82 (71-86) | 82 (76-87) | N/A | N/A | N/A | N/A | N/A | N/A |
| 6 | Kobayashi et al., 2018 | Retrospective  Cohort | 22 | 210 | 10 (45.5%) | 115 (54.8%) | 83 (71-88) | 83 (75-86) | 26 (25-27) | 27 (24-30) | N/A | N/A | 7.9 (6.1-12.3) | 6.6 (4.6-10.1) |
| 7 | Ochiai et al., 2020 [(17)](https://www.zotero.org/google-docs/?AHgzvk) | Prospective Cohort | 196 | 1230 | N/A | | N/A | | N/A | | N/A | | N/A | |
| 85 | Rylski et al., 2014 [(18)](https://www.zotero.org/google-docs/?uvdlaF) | Retrospective Cohort | 98 | 357 | 70 (71.4%) | 158 (44.3%) | 85.0 (9.0) | 85.2 (8.6) | N/A | | **NYHA Class I**:  0 (0%)  **NYHA Class II:** 5 (5.1%)  **NYHA Class III**: 51 (52.0%)  **NYHA Class IV:** 42 (42.9%) | **NYHA Class I**:  3 (0.8%)  **NYHA Class II:** 24 (6.7%)  **NYHA Class III:** 192 (53.8%)  **NYHA Class IV**: 133 (37.3%) | **High risk**  10 (4.8%) | **High risk**  11 (4.5%) |
|  |  |  |  |  |  |  |  |  |  |  |  |  |  |  |
| 9 | Ugwu et al., 2024 | Retrospective Cohort | 910 | 28.607 | 551 (71.2%) | 565 (73.0%) | 80.1 ± 8.7 | 80.4 ± 8.1 | N/A | N/A | N/A | N/A | N/A | N/A |
| 10 | Yu & Wang, 2023 [(19)](https://www.zotero.org/google-docs/?OxnOjw) | Retrospective Cohort | 73 | 166 | 207 (56.4%) | 86 (51.8%) | 73.7 ± 7.3 | 73.1 ± 7.3 | 24.2 ± 3.1 | 25.2 ± 3.8 | **NYHA Class III-IV**  52 (71.2%) | **NYHA Class III-IV**  115 (69.3%) | 6.0 (5.0–7.0) | 6.0 (5.0–7.0) |
|  |  |  |  |  |  |  |  |  |  |  |  |  |  |  |

**Supplementary Table 4.** Comorbidities of Patients in Included Studies

| **No.** | **Author, Year** | **Comorbidities** | | | | | | | | | | | | | | | | **Condition of Aortic Valve** | | | |
| --- | --- | --- | --- | --- | --- | --- | --- | --- | --- | --- | --- | --- | --- | --- | --- | --- | --- | --- | --- | --- | --- |
|  |  | **Smoking** | | **Hypertension** | | **Diabetes Mellitus** | | **Dyslipi**  **demia** | | **Coronary Artery Disease** | | **Atrial Fibrillation** | | **Peripheral Artery Disease** | | **Cerebrovascular Disease** | | **Bicuspid Aortic Valve** | | **Moderate-**  **Severe Aortic Regurgitation** | |
|  |  | **AAD** | **Non-**  **AAD** | **AAD** | **Non-**  **AAD** | **AAD** | **Non-**  **AAD** | **AAD** | **Non-**  **AAD** | **AAD** | **Non-**  **AAD** | **AAD** | **Non-**  **AAD** | **AAD** | **Non-**  **AAD** | **AAD** | **Non-**  **AAD** | **AAD** | **Non-**  **AAD** | **AAD** | **Non-**  **AAD** |
| 1 | An et al., 2023 | 33 (33.0%) | 118 (32.2%) | 48 (48.0%) | 237 (64.6%) | 16 (16.0%) | 104 (28.3%) | 72 (72.0%) | 292 (79.6%) | 32 (32.0%) | 189 (51.5%) | N/A | | 27 (27.0%) | 122 (33.2%) | 17 (17.0%) | 61 (16.6%) | 61 (61.0%) | 77 (21.0%) | (22.0%) | 89 (24.3%) |
| 2 | Boxhammer et al., 2023 | 4 (14.6%) | 3 (11.2%) | 21 (65.6%) | 2 (78.1%) | 2 (6.3%) | 10  (31.3%) | N/A | | 0 (0%) | 2 (6.3%) | 18 (56.3%)  : | 13 (40.6%) | N/A | | 1 (3.1%) | 2 (6.3%) | N/A | | N/A | |
| 3 | Fan et al., 2024 | 13 (26.5%) | 7 (11.9%) | 23 (46.9%) | 28 (45.1%) | 9 (18.3%) | 17 (27.5%) | N/A | | 37 (75.5%) | 44 (71%) | 11 (22.4%) | 7 (11.2%) | N/A | | 2 (4.1%) | 5 (8.1%) | 49  (100%) | 62  (100%) | 15 (30.6%) | 17 (27.4%) |
| 4 | Feng et al., 2024 | 51 (47.7%) | 170 (37.9%) | 53 (49.5%) | 294 (65.5%) | 18 (16.8%) | 117 (26.1%) | 58 (54.2%) | 291 (64.8%) | 25 (23.4%) | 212 (47.2%) | 20 (18.7%) | 74 (16.5%) | : 5 (4.7%) | 86 (19.2%) | 14 (13.1%) | **5**4 (12.0%) | 70 (65.4%) | 113 (25.2%) | N/A | N/A |
| 5 | Kassis et al., 2018 | N/A | N/A | 1.442 (86%) | 143.683 (84.9%) | 344 (20.5%) | 59,278 (35%) | N/A | N/A | 44 (2.9%) | 4.924 (2.9%) | N/A | N/A | N/A | N/A | 37 (2.2%) | 3.529 (2.1%) | 156 (9.3%) | 1.450 (0.9%) | N/A | N/A |
| 6 | Kobayashi et al., 2018 | 14 (63.6%) | 90 (42.9%) | 21 (95.4%) | 200 (95.2%) | 4 (18.2%) | 80 (38.1%) | 14 (63.6%) | 163 (77.6%) | 14 (63.6%) | 140 (66.7%) | N/A | N/A | N/A | N/A | 6 (27.3%) | 35 (16.7%) | 3 (13.6%) | 9 (4.3%) | 6 (27.2%) | 61 (29%) |
| 7 | Ochiai et al., 2020 | N/A | | N/A | | N/A | | N/A | | N/A | | N/A | | N/A | | N/A | | N/A | | N/A | |
| 8 | Rylski et al., 2014 | 20 (4%) | 79 (17%) | 79 (17%) | 265 (58%) | 108 (23%) | **2**8 (6%) | 68 (15%) | 227 (49%) | 53 (11%) | 137 (30%) | N/A | | 38 (8%) | 131 (28%) | N/A | | N/A | | N/A | |
| 9 | Ugwu et al., 2024 | 314 (40.6%) | 302 (39.0%) | 650 (84.0%) | 668 (86.3%) | 203 (26.2%) | 216 (27.9%) | N/A | | 592 (76.5%) | 598 (77.3%) | N/A | | N/A | | N/A | | N/A | | N/A | |
| 10 | Yu & Wang, 2023 | N/A | | 41 (56.2%) | **1**02 (61.4%) | 17 (23.3%) | 41 (24.7%) | 31 (42.5%) | 68 (41%) | 24 (32.9%) | 77 (46.4%) | 12 (16.4%) | 21 (12.7%) | 6 (8.2%) | 27 (16.3%) | N/A | | N/A | | 22 (30.1%) | 44 (26.5%) |

**Supplementary Table 5.** Forrest Plot for Post-Procedural LVEF

**
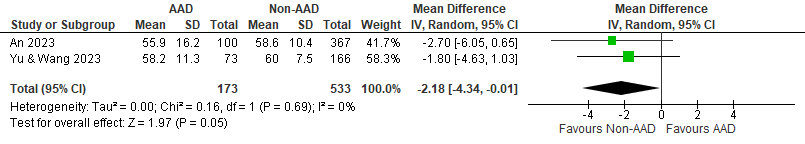
**

**Supplementary Table 6.** Peri- and Post- Procedural Characteristics

| **No** | **Author, Year** | **Echocardiographic Parameter** | | | | | | | | **Ascending aorta diameter based on CT Scan** | | **Follow up duration** | | **Type of TAVI Valve** | | **Mortality** | |
| --- | --- | --- | --- | --- | --- | --- | --- | --- | --- | --- | --- | --- | --- | --- | --- | --- | --- |
|  |  | **LVEF (%)** | | **Transaortic gradient (mmHg)** | | **Aortic valve area (cm2)** | | **Ascending aorta diameter (mm)** | |  |  |  |  |  |  |  |  |
|  |  | **AAD** | **Non-AAD** | **AAD** | **Non-AAD** | **AAD** | **Non-AAD** | **AAD** | **Non-AAD** | **AAD** | **Non-AAD** | **AAD** | **Non-AAD** | **AAD** | **Non-AAD** | **AAD** | **Non-AAD** |
| 1 | An et al., 2023 | **Initial**  60 (43.5–65)  **Post-**  **Procedural**  60 (47.3–65) | **Initial**  60 (51–65)  **Post-Procedural**  60 (55–65) | **Initial**  88.4 (74.0–108.2)  **Post-**  **Procedural**  21.2 (17.6–30.9) | **Initial**  88.4 (74.0–108.2)  **Post-Procedural**  23.0 (16.0–29.2) | N/A | N/A | **Initial**  45 42–48)  **Follow-up**  46 (44–48) | **Initial**  35 (32-38)  **Follow-up**  35 (31-38) | N/A | N/A | 19 (16–34) months | 27 (15–37) months | **BEV**  9 (9.0%)  **SEV**  91 (91.0%) | **BEV**  53 (14.4%)  **SEV**  314 (85.6%) | **In-hospital Mortality**  0 (0%)  **All-Cause Mortality**  0 (0.0%) | **In-hospital Mortality**  5 (1.4%)  **All-Cause Mortality**  5 (1.4%) |
| 2 | Boxhammer et al., 2023 | **Initial**  52.41 ± 11.53 | **Initial**  56.63 ± 12.15 | 46.44 ± 10.01 | 49.39 ± 13.93 | N/A | N/A | N/A | N/A | **Initial**  42.24 ± 2.19 | **Initial**  34.99 ± 2.48 | N/A | N/A | N/A | N/A | **1-year Mortality**  10 (31.2%)  **3-year Mortality**  15 (45.2%)  **5-year Mortality**  11 (36.0%) | **1-year Mortality**  12 (37.5%)  **3-year Mortality**  17 (54.8%)  **5-year Mortality**  13 (40.0%) |
| 3 | Fan et al., 2024 | **Initial**  55.6 ± 12.0 | **Initial**  56.0 ± 12.9 | **Initial**  59.2 ± 28.5 | **Initial**  90.1 ± 29.0 | **Initial**  517.3 ±  108.5 | **Initial**  472 ± 11.6 | N/A | | **Initial**  43.3 ± 2.8 | **Initial**  35.9 ± 3.8 | 54.1 ± 23.9 months | 59.3 ± 17. months | All patient used SEV | | **In-hospital mortality**  2(4.2%)  **All-cause mortality**  14(28.5%)  **Cardiovascular mortality**  5(10.2%) | **In-hospital mortality**  1(1.6%)  **All-cause mortality**  7(11.2%)  **Cardiovascular mortality**  3(4.8%) |
| 4 | Feng et al., 2024 | **Initial**  53.9 ± 14.5 | **Initial**  55.6 ± 13.3 | **Initial**  57.6 ± 19.5 | **Initial**  55.8 ± 17.1 | N/A | N/A | N/A | N/A | **Initial**  47.8 (46.5–50.4)  **Follow-up**  47.9 (46.2–50.6) | **Initial**  38.0 (34.7–40.5)  **Follow-up**  37.8 (35.2–40.9) | 1.9 (1.1 - 2.7) years | 1.3 (1.0-2.2) years | **BEV**  100 (93.5%)  **SEV**  7 (6.5%) | **BEV**  409 (92.5%)  **SEV**  33 (7.5%) | **In-hospital mortality**  3 (2.8%)  **All-cause mortality**  9 (8.9%)  **4-year Mortality**  5 (5.1%) | **In-hospital mortality**  5 (1.1%)  **All-cause mortality**  89 (19.9%)  **4-year Mortality**  39 (8.7%) |
| 5 | Kassis et al., 2018 | N/A | N/A | N/A | N/A | N/A | N/A | N/A | N/A | N/A | N/A | N/A | N/A | 156 (9.3%) | 1,450 (0.9%) | **In-hospital mortality**  43 (2.6%) | **In-hospital mortality**  4.217 (2.5%) |
| 6 | Kobayashi et al., 2018 | **Initial**  55 (50-60) | **Initial**  55 (45-60) | **Initial**  40 (37–45) | **Initial**  41 (34–51) | N/A | N/A | Major annulus  26 (23-27)  Minor annulus  21 (19-22) | Major annulus  26 (24–29)  Minor annulus  22 (20-23) | N/A | N/A |  |  | 3 (13.6%) | 9 (4.3%) | **In-hospital mortality**  1 (4.5%)  **6-months mortality**  2 (9.1%) | **In-hospital mortality**  12 (5.7%)  **6-months mortality**  16 (7.6%) |
| 7 | Ochiai et al., 2020 | **Initial**  54.4 ± 15.3 | **Initial**  57.8 ± 14.5 | 41.3 ± 13.8 | 43.5 ± 13.5 | 54.0 (42.0–67.0) | 50.0 (41.0–63.3) | N/A | N/A | N/A | N/A | 391 (99 to 727) days. | | N/A | N/A | **30 days mortality**  Not significant but no data presented  **2 year all-cause mortality**  70 (34.5%) | **30 days mortality**  Not significant but no data presented  **2 year all-cause mortality**  251 (20.4%) |
| 8 | Rylski et al., 2014 | **Initial**  60 ± 25 | **Initial**  60 ± 20 | 46 ± 15 | 46 ± 16 | 0.7 ± 0.2 | 0.5 ± 0.2 | 4.1 ± 0.2 | 3.1 0.5 | N/A | N/A | 14 months | | N/A | N/A | **In-hospital mortality**  7 (7.1%)  **1-year mortality**  10 (13.3%) | **In-hospital mortality**  17 (4.8%)  **1-year mortality**  41(16.9%) |
| 9 | Ugwu et al., 2024 | N/A | N/A | N/A | N/A | N/A | N/A | N/A | N/A | N/A | N/A | In-hospital  AAD: 4 (2.0-7.0) days  Non-AAD: 3 (2.0-6.0) days | | N/A | N/A | N/A | **In-hospital mortality**  16 (2.0%) |
| 10 | Yu & Wang, 2023 | **Post-**  **Procedural**  60.0 (46.5–65.0) | **Post-**  **Procedural**  60.0 (50.0–65.0) | 54.0 (42.0–67.0) | 50.0 (41.0–63.3) | N/A | N/A | 44.0 (42.0–46.0) | 36.0 (34.0–37.0) | 44.0 (42.0–46.0) | 36.0 (34.0–37.0) | 588 (384-1014) days | | **BEV**  8 (11%)  **SEV**  65 (89%)  **Balloon predilatation**  63 (86.3%)  **Balloon post-dilatation**  17 (23.3%) | **BEV**  19 (11.4%)  **SEV**  147 (88.6%)  **Balloon predilatation**  149 (89.8%)  **Balloon post-dilatation**  20 (12%) | *All-cause mortality*  ***Periprocedural (30 days)***  1(1.4%)  ***Early (1 year)***  3 (5.0%)  ***Late (3 years)***  4 (9.5%)  *Cardiovascular mortality*  ***Periprocedural (30 days)***  0 (0.0%)  ***Early (1 year)***  1(2.0%)  ***Late (3 years)***  1 (1.9%)  *Valve related mortality*  ***Periprocedural (30 days)***  0 (0.0%)  ***Early (1 year)***  0 (0.0%)  ***Late (3 years)***  0 (0.0%) | *All-cause mortality*  ***Periprocedural (30 days)***  4 (2.0%)  ***Early (1 year)***  15 (9.2%)  ***Late (3 years)***  18 (13.1%)  *Cardiovascular mortality*  ***Periprocedural (30 days)***  3 (1.8%)  ***Early (1 year)***  12 (7.7%)  ***Late (3 years)***  13 (10.0%)  *Valve related mortality*  ***Periprocedural (30 days)***  1 (0.6%)  ***Early (1 year)***  3 (1.9%)  ***Late (3 years)***  3 (1.9%) |

**Supplementary Table 7.** Forrest Plot for Post-Procedural Mortality

**Supplementary Table 7a. Forrest Plot for Peri-Procedural Mortality**

**
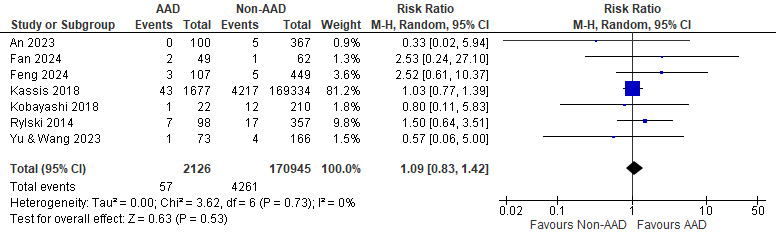
**

**Supplementary Table 7b. Forrest Plot for 1-year Mortality**

**
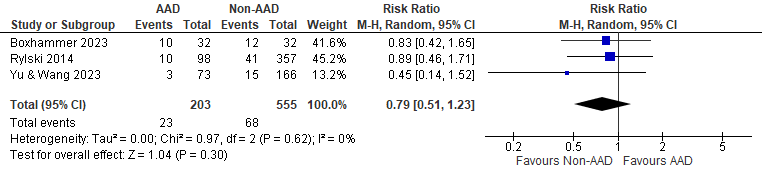
**

**Supplementary Table 8.** Post-procedural Complications

| **No** | **Author, Year** | **Conversion to open surgery** | | **Second device implantation** | | **Paravalvular regurgitation** | | **Aortic dissection** | | **Myocardial infarction** | | **Major Vascular Complication** | | **Permanent Pacemaker Implantation** | |
| --- | --- | --- | --- | --- | --- | --- | --- | --- | --- | --- | --- | --- | --- | --- | --- |
|  |  | **AAD** | **Non-AAD** | **AAD** | **Non-AAD** | **AAD** | **Non-AAD** | **AAD** | **Non-AAD** | **AAD** | **Non-AAD** | **AAD** | **Non-AAD** | **AAD** | **Non-AAD** |
| 1 | An et al., 2023 | 2 (2.0%) | 6 (1.6%) | 10 (10.0%) | 42 (11.4%) | **Initial**  22 (22.0%)  **Post-**  **Procedural**  5 (5.0%) | **Initial**  89 (24.3%)  **Post-**  **Procedural**  14 (3.8%) | 0 (0%) | 2 (0.5%) | 0 (0.0%) | 1 (0.3%) | 2 (2.0%) | 11 (3.0%) | 13 (13.0%) | 32 (8.7%) |
| 2 | Boxhammer et al., 2023 | N/A | N/A | N/A | N/A | N/A | N/A | N/A | N/A | N/A | N/A | N/A | N/A | N/A | N/A |
| 3 | Fan et al., 2024 | N/A | N/A | N/A | N/A | N/A | N/A | 0 (0.0%) | 1 (1.6%) | 1 (2%) | 2 (3.2%) | 0 (0.0%) | 0 (0.0%) | 2 (4.1%) | 9 (14.5%) |
| 4 | Feng et al., 2024 | 5 (4.7%) | 6 (1.3%) | 14 (13.1%) | 46 (10.5%) | 3 (2.8%) | 8 (1.8%) | 2 (1.9%) | 2 (0.4%) | 1 (0.9%) | 2 (0.4%) | 3 (2.8%) | 17 (3.8%) | 5 (4.7%) | 37 (8.2%) |
| 5 | Kassis et al., 2018 | N/A | N/A | N/A | N/A | N/A | N/A | 17 (1%) | 701 (0.4%) | 44 (2.9%) | 3.924 (2.9%) | N/A | N/A | N/A | N/A |
| 6 | Kobayashi et al., 2018 | N/A | N/A | N/A | N/A | N/A | N/A | N/A | N/A | 0 (0.0%) | 2 (1.0%) | 2 (9.1%) | 22 (10.5%) | 2 (9.1%) | 23 (11.0%) |
| 7 | Ochiai et al., 2020 | N/A | N/A | **7 (**3.6%) | 12 (1.0%) | 93 (47.4%) | 372 (30%) | N/A | N/A | N/A | N/A | N/A | N/A | N/A | N/A |
| 8 | Rylski et al., 2014 | N/A | N/A | N/A | N/A | N/A | N/A | N/A | N/A | N/A | N/A | N/A | N/A | N/A | N/A |
| 9 | Ugwu et al., 2024 | N/A | N/A | N/A | N/A | N/A | N/A | N/A | N/A | N/A | N/A | N/A | N/A | 70 (9.0%) | 61 (7.8%) |
| 10 | Yu & Wang, 2023 | 1 (1.4%) | 6 (3.6%) | 10 (13.7%) | 30 (18.1%) | 4 (5.5%) | 6 (3.6%) | 0 (0%) | 0 (0%) | 1 (1.4%) | 5 (3%) | N/A | N/A | 6 (8.2%) | 12 (7.2%) |

**Supplementary Table 9.** Forrest Plot for Post-Procedural Paravalvular Regurgitation

**
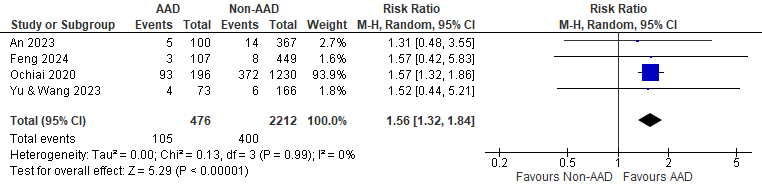
**

**Supplementary Table 10.** Forrest Plot for Post-Procedural Myocardial Infarction

**
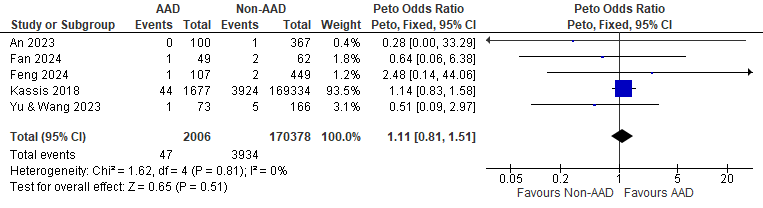
**

**Supplementary Table 11.** Forrest Plot for Post-Procedural Permanent-Pacemaker Placement

**
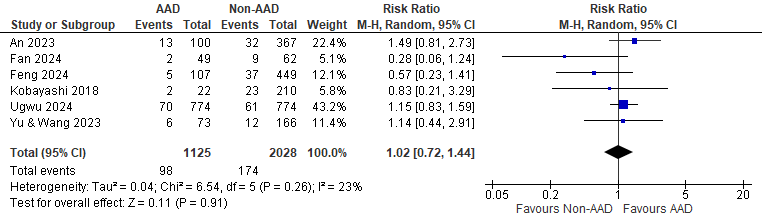
**

**Supplementary Table 12.** Forrest Plot for Post-Procedural Aortic Dissection

**
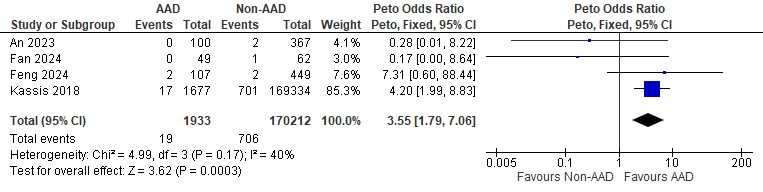
**

**Supplementary Table 13.** Forrest Plot for Post-Procedural Conversion to Open Surgery

**
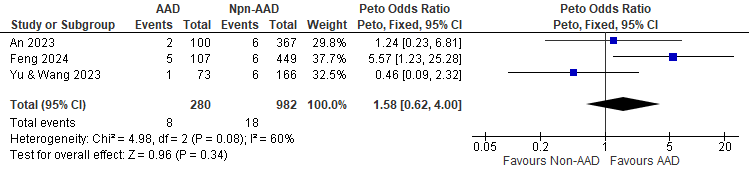
**

**Supplementary Table 14.** Forrest Plot for Post-Procedural Second Valve Implantation

**
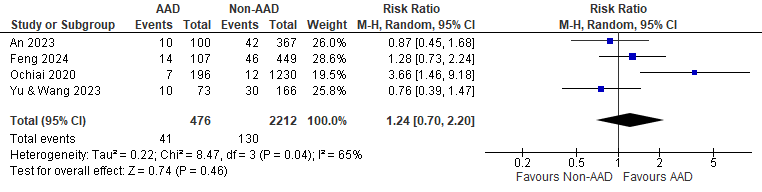
**
